# Growth Anomalies on the Coral Genera *Acropora* and *Porites* Are Strongly Associated with Host Density and Human Population Size across the Indo-Pacific

**DOI:** 10.1371/journal.pone.0016887

**Published:** 2011-02-18

**Authors:** Greta S. Aeby, Gareth J. Williams, Erik C. Franklin, Jessica Haapkyla, C. Drew Harvell, Stephen Neale, Cathie A. Page, Laurie Raymundo, Bernardo Vargas-Ángel, Bette L. Willis, Thierry M. Work, Simon K. Davy

**Affiliations:** 1 Hawaii Institute of Marine Biology, Kaneohe, Hawaii, United States of America; 2 School of Biological Sciences, Victoria University of Wellington, Wellington, New Zealand; 3 Center for Marine Biodiversity and Conservation, Scripps Institution of Oceanography, La Jolla, California, United States of America; 4 ARC Centre of Excellence for Coral Reef Studies, and School of Marine and Tropical Biology, James Cook University, Townsville, Queensland, Australia; 5 Department of Ecology and Evolutionary Biology, Cornell University, Ithaca, New York, United States of America; 6 Australian Institute of Marine Science, Townsville, Queensland, Australia; 7 University of Guam Marine Lab, University of Guam (UOG) Station, Mangilao, Guam; 8 University of Hawaii, Joint Institute for Marine and Atmospheric Research, Honolulu, Hawaii, United States of America; 9 U. S. Geological Survey, National Wildlife Health Center, Honolulu Field Station, Honolulu, Hawaii, United States of America; King Abdullah University of Science and Technology, Saudi Arabia

## Abstract

Growth anomalies (GAs) are common, tumor-like diseases that can cause significant morbidity and decreased fecundity in the major Indo-Pacific reef-building coral genera, *Acropora* and *Porites.* GAs are unusually tractable for testing hypotheses about drivers of coral disease because of their pan-Pacific distributions, relatively high occurrence, and unambiguous ease of identification. We modeled multiple disease-environment associations that may underlie the prevalence of *Acropora* growth anomalies (AGA) (n = 304 surveys) and *Porites* growth anomalies (PGA) (n = 602 surveys) from across the Indo-Pacific. Nine predictor variables were modeled, including coral host abundance, human population size, and sea surface temperature and ultra-violet radiation anomalies. Prevalence of both AGAs and PGAs were strongly host density-dependent. PGAs additionally showed strong positive associations with human population size. Although this association has been widely posited, this is one of the first broad-scale studies unambiguously linking a coral disease with human population size. These results emphasize that individual coral diseases can show relatively distinct patterns of association with environmental predictors, even in similar diseases (growth anomalies) found on different host genera (*Acropora* vs. *Porites*). As human densities and environmental degradation increase globally, the prevalence of coral diseases like PGAs could increase accordingly, halted only perhaps by declines in host density below thresholds required for disease establishment.

## Introduction

Coral reefs represent some of the most biologically diverse ecosystems on the planet, but these important habitats are declining worldwide due to human overexploitation, land-based pollution, global climate change, and disease outbreaks [1]–[6]. While the situation is most severe in the Caribbean, coral reefs are also in decline across the Indo-Pacific, where an annual loss in coral cover of approximately 1% has occurred over the last 20 years, increasing to 2% between 1997 and 2003 [7]. Coral diseases contribute to this decline by causing a loss of live coral cover [8]–[10] that, under extreme circumstances, can lead to complete community phase-shifts (e.g. from coral-dominated to alga-dominated) [11]. The causes of most coral diseases are unknown. However, understanding how coral disease prevalence relates to changes in reef environmental quality may provide clues to disease etiology. Coral disease increases are associated with local anthropogenic stressors such as poor water quality [12]–[17], as well as global stressors such as sea-surface temperature anomalies [18] and resultant coral bleaching events [19]–[22]. Effects of environmental co-factors may vary between disease types [23] but few efforts have been made to model individual coral diseases with multiple, possibly interacting, environmental cofactors, but see [17], [18], [22].

**Figure 1:**
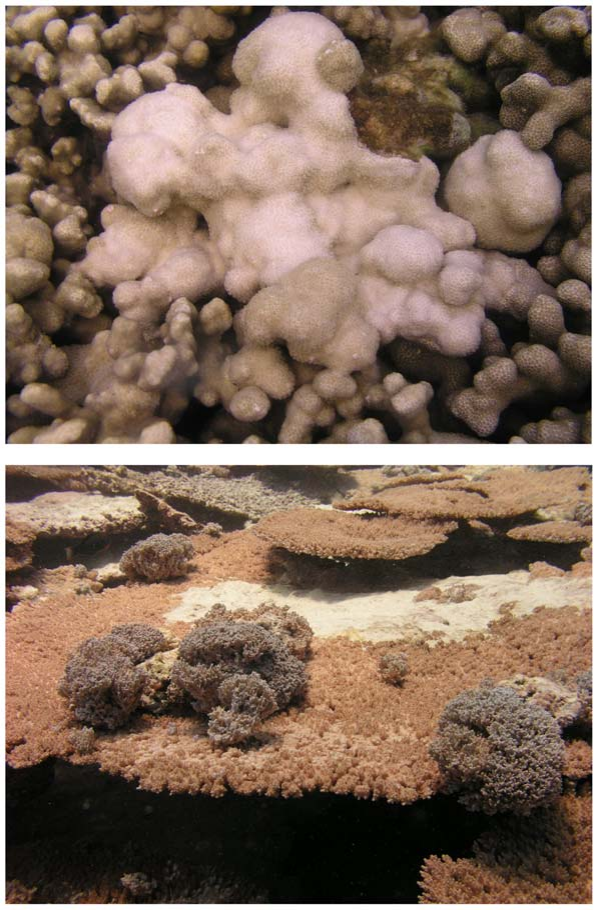
Picture of *Porites* growth anomaly (top) and *Acropora* growth anomaly (bottom).

As a step towards understanding disease dynamics, statistical modeling techniques have recently been used over small spatial scales (individual reefs) to examine multiple coral disease-environment associations [17]. In the present study we used statistical modeling to examine the prevalence of two coral diseases, *Acropora* growth anomalies (AGAs) and *Porites* growth anomalies (PGAs) (Fig. 1) across the Indo-Pacific region. Growth anomalies appear as distinctive protuberant masses on corals and thus are easily distinguished in the field. These lesions do not suffer from confounding interpretations, as do lesions involving tissue loss (e.g. white syndrome), which may be confused with predation or vice versa. Growth anomalies have been reported to affect a variety of coral genera from both the Caribbean and the Indo-Pacific [24], [25] and have been relatively well characterized at the gross and microscopic levels [26]–[34]. Although the causes of GAs in corals are unknown, they are associated with reduced colony growth [26], [27], partial colony mortality [28], [33] and decreased reproduction [30], [33], and therefore could negatively impact the fitness of host populations. Acroporids appear to be the most susceptible to GAs; they have been recorded in over 17 species [25], [28], [33], [34]. *Porites* GAs are less common and have been described from seven species [22], [25], [32], [34], [35].

Our objective was to model the prevalence of growth anomalies in *Porites* spp. and *Acropora* spp. in relation to a range of environmental parameters at several hundred sites across the Indo-Pacific Ocean. Disease data were collected from reefs in regions that ranged from heavily populated (and therefore potentially more intensely impacted by local stressors), such as the main Hawaiian Islands [36] and Central Philippines [37], to relatively pristine remote reefs with minimal direct human impact, although still vulnerable to global stressors, such as Palmyra Atoll National Wildlife Refuge in the northern Line Islands [38], [39]. This enabled comparative analyses of disease prevalence across multiple gradients for each of our predictors of interest: biological factors (coral host abundance); anthropogenic factors (human population size); and environmental factors (thermal stress events, surface ultra-violet radiation). Our overall aim was to determine the environmental conditions associated with the prevalence of AGAs and PGAs across the Indo-Pacific, while accounting for confounding effects such as variations in survey effort and timing of the disease surveys.

## Methods

### Prevalence of Acropora and Porites growth anomalies, and potential biological, environmental and anthropogenic predictors

**Figure 2:**
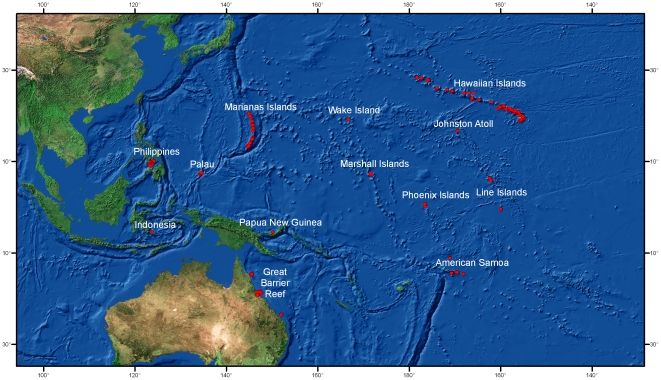
Map showing survey sites across the Indo-Pacific used in the analyses.

Our analyses were based upon 937 quantitative coral disease surveys from 13 regions from across the Indo-Pacific between 2002 and 2008 (Fig. 2; Table 1; Table S1). Our response variable was disease prevalence (proportion of colonies surveyed affected by GAs) within the survey areas. Biological predictors were host (*Porites* spp. or *Acropora* spp.) density and percent cover.

**Table 1:** Numbers of disease surveys conducted at each region by year.

Survey region	2002	2004	2005	2006	2007	2008	Total
Great Barrier Reef		38	42	36	6	12	**134**
Papua New Guinea			4				**4**
Indonesia			5		5		**10**
Philippines				22	11		**33**
American Samoa		11	19	57		58	**145**
Palau		6	19				**25**
Marshall Islands		4					**4**
Marianas				7	66		**73**
Line Islands				36		46	**82**
Phoenix Islands				12		8	**20**
Johnston Atoll		12		25		6	**43**
Wake					12		**12**
Hawaiian Islands	57	82	100	113			**352**
							
					**Total**		**937**

Belt transects were used to quantify disease and biological predictors, but the number, length and width of transects differed among regions and researchers. Hence, both survey effort (area of reef surveyed (m^2^)) and timing of the surveys (year) were included as predictors in the models. Global environmental predictors included frequency of weekly sea surface temperature anomalies (WSSTA) and frequency of erythemal surface ultraviolet (UV) radiation anomalies, while human population size served as a proxy for the impact of anthropogenic effects. Coral disease survey locations were imported as geo-referenced points into the GIS and predictor values were extracted for each survey. Human population counts were raster data of 2.5 arc-minutes resolution adjusted to match UN totals for 2005 [40]. Human population size was summed within circular buffers of 1 and 100 km around each survey site. Data were included for all grid cells that intersected a buffer. The mean annual WSSTA values for the four years prior to the year of the survey were extracted for each coral survey location. The frequency of weekly sea surface temperature anomalies (WSSTA) was defined as the number of times over the previous 52 weeks that the weekly sea surface temperature (SST) minus the weekly climatological SST, equaled or exceeded 1°C [41]. SSTA data were approximately 4 km resolution Pathfinder AVHRR raster data on a weekly time scale from 1985 through 2005. The frequency of erythemal surface ultraviolet (UV) radiation anomalies were the number of times between 2000 and 2004 that the monthly average exceeded the climatological mean plus one standard deviation [42]. These values were summed across the 12 months to provide a single value, ranging from 0–19, representing the number of anomalous values for each coral survey location over the entire 5 years. The erythemal surface UV data were measured as part of the GSFC TOMS EP/TOMS satellite program at NASA [43]. These data were processed by NASA to isolate the amount of erythemal ultraviolet (UV) light that reaches Earth's surface. Data were reported as the average Joules (J) per m^2^ per month at one-degree cell (∼110 km by 110 km) resolution. Figure S1 shows how GIS data were used in the analyses for the main Hawaiian Islands, as an example. These data were prepared and geoprocessed with ArcGIS 9.2 and Matlab 7.1.

#### Statistical analyses

To investigate associations between prevalence (proportion of colonies affected by GAs) of AGAs and PGAs with each of the predictor variables (Table 2), we used a permutational distance-based multiple regression technique (DISTLM) [44]. DISTLM is robust to zero-inflated data sets, such as ours, and makes no assumptions about the distribution of the response variable (i.e. normality does not have to be satisfied). No two predictors exceeded the recommended cut-off inter-correlation value of 0.95 [45]. In fact, the highest Pearson's correlations between predictors did not exceed 0.65 and 0.44 for AGA and PGA, respectively. Predictors were normalized and were fitted conditionally in a step-wise manner, with tests based on 9999 permutations of the residuals under the reduced model [44], [45]. Model selection (to obtain the best-fit model while maintaining model parsimony) was based on a Bayesian Information Criterion (BIC) [46]. BIC is similar to the more commonly used Akaike's Information Criterion (AIC), however BIC includes a more severe penalty for the inclusion of extraneous predictor variables [45]. To visualize each best-fit model, distance-based redundancy plots (dbRDA) [44] were created based on the prevalence patterns between independent observations. The optimal predictor variable vector(s) (model base variables) was then overlaid as a bi-plot [45]. DISTLM cannot handle missing values within the predictor variable data sets, therefore disease surveys with missing data points for any of the nine predictor variables had to be deleted from the analyses, leaving 304 and 602 surveys for AGA and PGA prevalence, respectively. All prevalence modeling analyses were based on zero-adjusted Bray-Curtis similarity matrices [47] and conducted in PRIMER v6 [48] and PERMANOVA+ [45].

**Table 2:** Response and predictor variables used in the analyses with their codes and units.

Variable	Code	Description and units	Min	Max
Response				
*Acropora* GA	AGA	prevalence	0	9.38
*Porites* GA	PGA	prevalence	0	16.67
Predictor				
*Acropora* cover	AcropCov	% cover	0.40	75.1
*Acropora* density	AcropDen	# colonies/m^2^	0.01	37.8
*Porites* cover	PorCov	% cover	0.2	90.8
*Porites* density	PorDen	# colonies/m^2^	0.03	41.1
Depth	Depth	m	0.5	18.3
WSSTA during prior 4 years	WSSTA	mean number	1.5	20
Human numbers within 1 km	HumPop1	number of people	0	50,362
Human numbers within 100 km	HumPop100	number of people	0	7,705,440
UV input	UV	rating scale	0	15
Year	Year	year of survey	2002	2008
Survey effort	Area	m^2^ of reef	60	1200

Min/Max, minimum and maximum predictor values between independent observations across the entire data set. GA, growth anomaly. WSSTA, weekly sea-surface temperature anomaly. UV, ultraviolet radiation.

## Results

**Figure 3:**
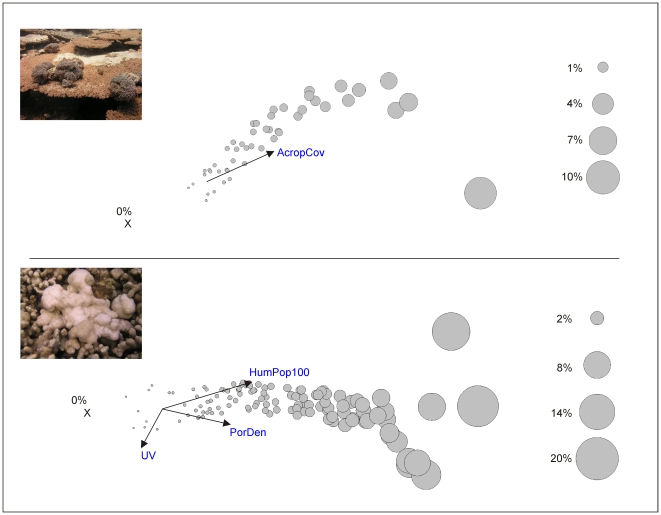
Distance-based multiple regression analyses relating *Acropora* (top) and *Porites* (bottom) growth anomaly prevalence to 9 predictor variables across surveys throughout the Indo-Pacific. Number of surveys where data for all predictor variables was obtained equals 304 and 602 for *Acropora* GAs and *Porites* GAs, respectively. Graphs modified from distance-based redundancy plots. The bubbles represent the proportion of corals displaying signs of the disease (% of the population affected) at each survey site. The overlaid bi-plot shows the correlation of the disease prevalence with the optimal predictor(s) forming the best-fit model. The vector line indicates the direction of the relationship with disease prevalence. The length of vector line indicates the relative importance of the predictor. X represents a cluster of sites where the disease prevalence equaled zero. Predictor variable codes and units are as per Table 2.

Between 2002 and 2008, AGAs were recorded within approximately 16% of the surveys (n = 534) and PGAs were recorded within 18% of the surveys (n = 855) (Table S2). Prevalence of AGAs (all years and surveys combined) ranged from 0 to 9.4% (Avg. = 0.14%, SD±0.6) and the prevalence of PGAs ranged from 0 to 16.7% (Avg. = 0.2% SD±1.1) (Table S3). AGA prevalence was positively associated with *Acropora* cover, which explained 16.6% of the variability in disease prevalence (Table 3). No other predictor explained a significant proportion of the variability in AGA prevalence (Table 3, Fig. 3). PGA prevalence was positively associated with higher regional (100 km) human population sizes and with higher *Porites* colony densities, with the two predictors significantly explaining 28.8% of the variability in disease prevalence. UV input also significantly explained 12.4% of the variability in disease prevalence and increased levels of UV were associated with lower levels of PGA prevalence (Table 3, Fig. 3). The nine predictors explained a greater proportion of the variability in PGA prevalence than in AGA prevalence, with total explained variability equaling 41.2% and 16.6%, respectively (Table 3).

**Table 3:** Summary results of a distance-based permutational multiple regression analysis for the association of the prevalence of two coral diseases (*Acropora* and *Porites* growth anomalies) with 9 predictor variables across surveys (304 and 602, respectively) throughout the Indo-Pacific Ocean.

Disease	n	Predictor	BIC	Pseudo-F	P value	% variability	% total
*Acropora* GA	304	AcropCov	1925.5	21.18	0.0001	16.6	16.6
*Porites* GA	602	HumPop100	4349.2	36.88	0.0001	15.8	
		PorDen	4335.9	19.98	0.0001	13.0	
		UV	4325.8	16.57	0.0002	12.4	41.2

The optimal predictors of each disease and the proportion of variability (%) in the data set they explained are shown. Predictor variable codes and units are as per Table 2. Model development was based on step-wise selection and a Bayesian Information Criterion (BIC), with the total variation (r^2^) explained by each best-fit model shown (% total). Analyses based on 9999 permutations of the residuals under a reduced model.

## Discussion

Growth anomalies (GAs) in *Acropora* (AGAs) and *Porites* (PGAs) were widespread across the Indo-Pacific occurring in eleven of the thirteen survey regions. GAs were relatively common with the overall frequency of occurrence (percentage of surveys containing GAs) across the Indo-Pacific being 16% for AGAs and 18% for PGAs. Some regions had an even higher disease occurrence, such as the Philippines where PGAs were found in 58% of the surveys (n = 33) and in Palau where AGAs were found in 32% (n = 25). In contrast to the Indo-Pacific, GAs are much less frequent within the Caribbean. For example, no GAs were reported from 160 stations surveyed across the Florida Keys [49], 13 reef areas off the coast of Colombia [50] and 23 sites off Mexico [51]. In fact, there have only been two published reports of AGAs from the Caribbean [27], [28] with no published reports of PGAs.

Although both diseases (AGA and PGA) were widespread on reefs throughout the Indo-Pacific, their average total prevalence was low (<1%). These values are consistent with reports of other diseases within the Indo-Pacific. For example, mean black band disease prevalence at 19 reefs across the GBR equaled 0.1% [52] and white syndrome and GA prevalence in southeast Sulawesi, Indonesia equaled 0.42% and 0.15%, respectively [53]. In Guam, total GA prevalence averaged 1.4% and that of skeletal eroding band, 1.2% [54], in American Samoa, the prevalence of 12 coral diseases was each less than 1% [55], and finally at Palmyra Atoll overall disease prevalence equaled less than 0.4% [56]. However, on some reefs within the Indo-Pacific coral diseases can be quite prevalent. Prevalence of skeletal eroding band from the reefs of Aqaba, Red Sea, ranged from 4 – 41% [57] and the average prevalence of *Porites* ulcerative white spot disease in the Philippines was 22% [58]. In Guam, white syndrome is, by far, the most prevalent disease (8.9%) [54] and this has remained consistent for several years (Raymundo and Kim *unpubl. data*). However, while these comparisons provide a snapshot view of regional variability, they do not take into account the possibility that some of these high values may represent seasonal outbreak conditions at surveyed sites and differences in the amount of reef area surveyed.

The emergence of coral disease occurs from a complex interplay between the host, causative agent and environment [23]. Hence, one would expect high variability between sites, as found in this and other studies of coral disease [8], [14], [17], [37], [38], [54], [59]–[61]. The prevalence of AGAs and PGAs varied greatly among survey sites and survey regions. The reefs within the regions we examined represented a range of environmental conditions, differing in water temperature, exposure to ultraviolet radiation, coral host abundance and human population sizes. Using statistical modeling, we found relatively distinct environmental associations between the prevalence of AGAs and PGAs throughout the Indo-Pacific. To sum, the prevalence of AGAs was most positively associated with host abundance, while PGA prevalence showed strong positive association with both increased human population sizes and host abundance. In addition, low prevalence of PGAs on reefs (as opposed to zero prevalence) was associated with increased frequencies of ultraviolet radiation anomalies. These results emphasize that individual coral diseases can show relatively distinct patterns of association with environmental predictors [17] even in the case of similar diseases (GAs) found on different host genera (*Acropora* vs. *Porites*). Therefore, future efforts to predict impacts and manage coral diseases on reefs should consider this finding and treat analyses separately for each disease, rather than combining all diseases into a single response variable.

Model performance was good for PGAs, with 41.1% of the variability in prevalence explained. Therefore, we predict that within the Indo-Pacific one would encounter PGAs on reefs with higher *Porites* cover near high human population centers. In contrast, less variability (16.6%) was explained by modeling AGAs, suggesting that additional variables we did not test may be implicated in driving prevalence patterns. For our analyses, disease data were collected at the genus level, which does not take into account potential species specific differences in susceptibility to GAs. For example, across the Indo-Pacific, the genus *Acropora* is very species-rich (>160 species) [62]. If species within the genus were differentially susceptible to AGAs then this could partially explain the poor model fit, as our taxonomic resolution did not account for host density differences below the genus level. The prevalence of AGAs in American Samoa, NWHI and Johnston Atoll was higher in plating *Acropora* sp. (n = 29) as compared to branching (n = 8), encrusting (n = 2) and corymbose (n = 15) morphologies, suggesting that plating colonies may be more prone to GA formation [33]. Many coral species are difficult to identify in the field but including information such as morphological types within genera during surveys may provide more resolution and better explain prevalence patterns.

While it is likely the performance of our models would be improved with species level data, we still found that generic host abundance was an important explanatory variable for the prevalence of both AGAs and PGAs. A positive association between a disease and its host is consistent with disease ecology theory [63], and often reflects the increased horizontal transmission of a disease throughout a population as the population increases in size and distance between individuals decreases [64], [65]. Many examples of relationships between host abundance and disease prevalence exist throughout a wide range of ecosystems and taxa, governed by both density-dependent and frequency-dependent processes [66]–[71]. Host abundance thresholds occur for other coral diseases; for example white syndrome outbreaks along the GBR require, in part, host cover values in excess of 50% [18]. On reefs in Guam and Palau, total disease prevalence was significantly positively associated with coral host abundance or cover [54] and, in Hawaii, *Porites* trematodiasis and *Montipora* white syndrome prevalence are both strongly associated with coral host cover [17], [72], [73]. Thus, diseases causing significant mortality and reduced fecundity are likely to have major effects on community structure, as spatially-dominant species will be more impacted by disease.

Only PGA prevalence, and not AGA, showed strong positive associations with human population size suggesting that they are related, directly or indirectly, to some environmental co-factor associated with increased human population size at regional spatial scales. Human activities can result in increased disease levels within wildlife populations, as a result of human-induced environmental degradation caused by pollution, eutrophication, habitat fragmentation, and direct introduction of novel pathogens into ecosystems [74]–[79]. For example, the Hawaiian green sea turtle showed elevated rates of a tumor disease in watersheds with a high nitrogen-footprint reflective of coastal eutrophication [80]. Diseases of corals in tropical ecosystems are proving no exception, with human impacts suggested to affect disease prevalence [81]. If we are to conserve our coral reef resources, it is critical that we determine which components of human impacts may be affecting disease levels. Increased nutrients and reduced water quality have been linked to increased prevalence and severity of coral diseases such as black band disease (caused by a microbial consortium) [13]–[15], [82], and aspergillosis, a sea fan disease caused by the terrestrial soil-borne fungus *Aspergillus sydowii* [12], [15], [16], [83], [84]. Direct influx of potential pathogens into the marine environment (e.g. through sewage effluent disposal), has been suggested as a causal mechanism for white pox which affects elkhorn *Acropora* corals in the Caribbean [85]. Although not well-studied, viruses have also been proposed as potential agents of coral disease [86] and marine virus-like particles (VLPs) have been found in increased abundance with proximity to populated coastal areas [87].

While our understanding of coral disease etiology has advanced considerably in recent years [88]–[93], the cause of coral GAs remains largely unknown [33]. For AGAs, damage to cells from ultraviolet (UV) radiation [29] and stressors such as high levels of sedimentation, turbidity and seasonal temperature extremes [28] have been suggested as playing a role in triggering GA formation. Our analyses suggest a link between PGA prevalence and ultraviolet radiation anomalies in areas where human population sizes are lower, however, no such associations were found for AGAs. The link between PGA development and ultraviolet radiation was not supported manipulatively on *Porites compressa* in Hawaii [94] and no explanations have yet been presented regarding the etiology of PGAs, but one study did find them to be transmissible suggesting an infectious agent [35]. Viruses have been found associated with tumor formation in other animals such as turtles [95], [96] and fish [97]. Given the known positive association between human numbers and densities of marine viruses [87], [98], the common association of viruses with the coral holobiont [99], [100] and the strong association we found between PGAs and human population size, investigations into a potential viral etiology of PGAs would seem a logical next step.

 Increases in temperature, like other stressors such as poor water quality, can alter host susceptibility to disease or pathogen virulence [6], [101], [102], ultimately shifting the balance in favor of one or the other [103]. Many coral diseases show positive associations with temperature, for example black band disease in the Caribbean, the Florida Keys and the GBR [104]–[107], *Porites* tissue loss syndrome in Hawaii [17], and white syndromes along the GBR [18]. However, we found that host abundance and human population size were the optimal predictors for variations in prevalence of AGAs and PGAs, respectively, with WSSTAs showing no such association. It may be that chronic diseases, such as GAs, are less influenced by short-term changes in temperature as compared to the tissue loss diseases, many of which are caused by pathogenic bacteria with virulence factors that may be enhanced at higher temperatures [85], [90], [91], [93]. Many bacteria thrive in warm temperatures and so bacterial diseases could be influenced more by temperature [108]. Understanding the disease-specific responses to environmental and anthropogenic stressors is critical if we are to protect and conserve our reefs from the inevitable threat of future environmental change.

In summary, AGAs and PGAs showed relatively distinct patterns with the predictors tested throughout the Indo-Pacific. While GAs in both genera showed positive associations with host abundance, PGAs additionally showed strong positive associations with human population size. GAs are often progressive and can result in host mortality [33] and so represent a threat to coral reef health worldwide. As human densities and environmental degradation continue to increase across the globe [78], the prevalence of diseases such as PGAs that are associated with these factors may similarly increase throughout the Indo-Pacific, halted only perhaps by declines in host density below thresholds required for disease establishment. Increases in coral disease prevalence and outbreaks, in combination with mass coral bleaching events and other disturbances associated with climate change, pose a great threat to the future survival of coral reef environments on our planet. Future efforts should focus on determining the etiology of AGAs and PGAs so that the environmental associations identified in the present study are put into a better ecological context, thus increasing our understanding of their ecology and ultimately granting us the knowledge to mitigate an increase in their prevalence.

## Supporting Information

Figure S1 **Example of GIS data used in the analyses.** Shown are data for the sites included from the main Hawaiian Islands.
(TIF)

Table S1 Islands surveyed for *Acropora* and *Porites* growth anomalies within each of the regions analyzed.
(DOC)

Table S2 Frequency of occurrence (FOC) of *Acropora* growth anomalies (AGAs) and *Porites* growth anomalies (PGAs) across the Indo-Pacific.
(DOC)

Table S3 Average prevalence of *Acropora* growth anomalies (AGAs) and *Porites* growth anomalies (PGAs) across the Indo-Pacific.
(DOC)
