# Supplementary figures and images for: Growth Anomalies on the Coral Genera Acropora and Porites Are Strongly Associated with Host Density and Human Population Size across the Indo-Pacific
Source: PLoS One. 2011 Feb 18;6(2):e16887. doi: 10.1371/journal.pone.0016887 (PMC3041824; doi:10.1371/journal.pone.0016887)

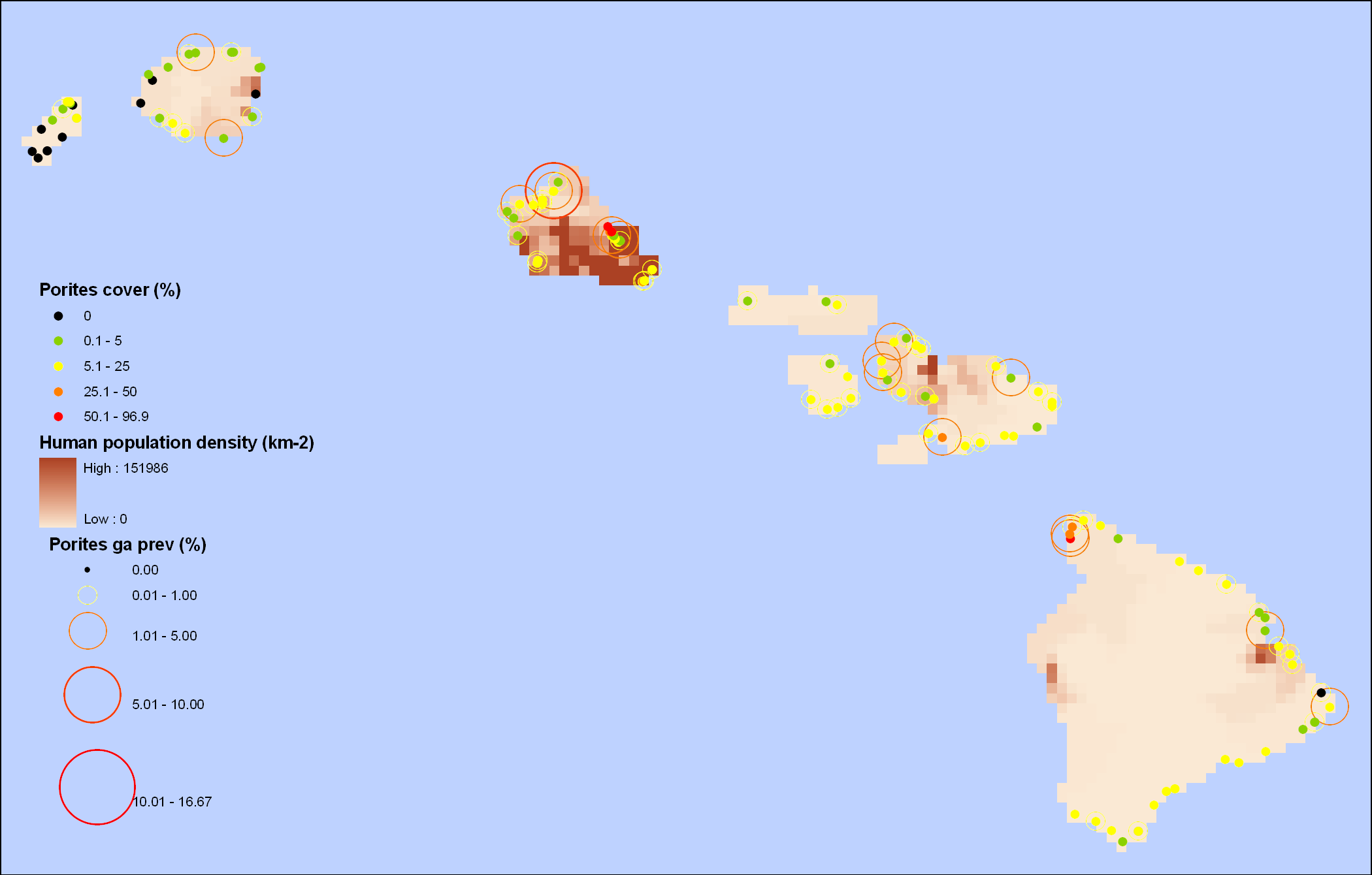

Supplement: Figure S1 — Example of GIS data used in the analyses. Shown are data for the sites included from the main Hawaiian Islands. (TIF) [file pone.0016887.s001.tif]
