# Supplementary material for: Growth Anomalies on the Coral Genera Acropora and Porites Are Strongly Associated with Host Density and Human Population Size across the Indo-Pacific
Source: PLoS One. 2011 Feb 18;6(2):e16887. doi: 10.1371/journal.pone.0016887 (PMC3041824; doi:10.1371/journal.pone.0016887)
Supplement: Table S1 — Islands surveyed for Acropora and Porites growth anomalies within each of the regions analyzed. (DOC) [file pone.0016887.s002.doc]

| **Survey region** | **Island** |
| --- | --- |
| **Great Barrier Reef** | One Tree island, Heron Island, Magnetic Island, |
|  | Orpheus Island, Lizard Island |
| **American Samoa** | Tutuila, Ofu-Olosega, Rose, Tau, Swains |
| **Wake** | Wake |
| **Palau** | Palau |
| **Marshall Islands** | Majuro |
| **Marianas Islands** | Guam, Saipan, Rota, Tinian, Sarigan, Alamagan |
|  | Agrihan, Maug, Uracas, Asunion, Pagan, Guguan |
| **Philippines** | Central Visayas |
| **Indonesia** | Sulawesi |
| **Papua New Guinea** | Papua New Guinea |
| **Johnston Atoll** | Johnston Atoll |
| **Hawaiian Islands** | *Main Hawawiian Islands* |
|  | Maui, Big Island, Kauai, Molokai, Kaula |
|  | Lanai, Lehua, Ni'ihau, Oahu |
|  | *Northwestern Hawaiian Islands* |
|  | French Frigate Shoals, Gardner, Maro, Laysan, Kure |
|  | Lisianski, Midway, Necker, Nihoa, Pearl & Hermes |
| **Phoenix Islands** | Howland |
|  | Baker |
| **Line Islands** | Palmyra |
|  | Kingman Reef |
|  | Jarvis |
