# Supplementary material for: Growth Anomalies on the Coral Genera Acropora and Porites Are Strongly Associated with Host Density and Human Population Size across the Indo-Pacific
Source: PLoS One. 2011 Feb 18;6(2):e16887. doi: 10.1371/journal.pone.0016887 (PMC3041824; doi:10.1371/journal.pone.0016887)
Supplement: Table S2 — Frequency of occurrence (FOC) of Acropora growth anomalies (AGAs) and Porites growth anomalies (PGAs) across the Indo-Pacific. (DOC) [file pone.0016887.s003.doc]

| **Survey region** | **# sites with AGA** | **# sites with *Acropora*** | **FOC AGA (%)** | **# sites with PGA** | **# sites with *Porites*** | **FOC PGA (%)** |
| --- | --- | --- | --- | --- | --- | --- |
| Great Barrier Reef | 37 | 134 | **27.6** | 18 | 134 | **13.4** |
| Papua New Guinea | 0 | 4 | **0** | 0 | 4 | **0** |
| Indonesia | 2 | 10 | **20** | 2 | 10 | **20** |
| Philippines | 0 | 33 | **0** | 19 | 33 | **57.6** |
| American Samoa | 19 | 123 | **15.4** | 9 | 136 | **6.6** |
| Palau | 8 | 25 | **32** | 8 | 25 | **32** |
| Marshall Islands | 1 | 4 | **25** | 0 | 4 | **0** |
| Marianas | 3 | 46 | **6.5** | 5 | 68 | **7.4** |
| Line Islands | 10 | 53 | **18.9** | 17 | 72 | **23.6** |
| Phoenix Islands | 1 | 19 | **5.3** | 0 | 17 | **0** |
| Johnston Atoll | 0 | 40 | **0** | 0 | 9 | **0** |
| Wake | 1 | 3 | **33.3** | 4 | 12 | **33.3** |
| Hawaiian Islands | 3 | 40 | **7.5** | 72 | 331 | **21.8** |
| **total** | 85 | 534 | **15.9** | 154 | 855 | **18** |
