# Supplementary material for: Growth Anomalies on the Coral Genera Acropora and Porites Are Strongly Associated with Host Density and Human Population Size across the Indo-Pacific
Source: PLoS One. 2011 Feb 18;6(2):e16887. doi: 10.1371/journal.pone.0016887 (PMC3041824; doi:10.1371/journal.pone.0016887)
Supplement: Table S3 — Average prevalence of Acropora growth anomalies (AGAs) and Porites growth anomalies (PGAs) across the Indo-Pacific. (DOC) [file pone.0016887.s004.doc]

| **Survey region** | **avg. prev AGA(%)** | **AGA range** | **avg. prev PGA (%)** | **PGA range** |
| --- | --- | --- | --- | --- |
| Great Barrier Reef | 0.091 | 0-1.7 | 0.299 | 0-7.5 |
| Papua New Guinea | 0 | 0 | 0 | 0 |
| Indonesia | 0.149 | 0-0.88 | 0.069 | 0-0.37 |
| Philippines | 0.152 | 0-5 | 0.851 | 0-14.1 |
| American Samoa | 0.216 | 0-9.4 | 0.047 | 0-2.1 |
| Palau | 0.14 | 0-1.4 | 0.19 | 0-1.8 |
| Marshall Islands | 0.055 | 0-0.22 | 0 | 0 |
| Marianas | 0.12 | 0-3.1 | 0.03 | 0-0.89 |
| Line Islands | 0.31 | 0-4.2 | 0.25 | 0-8.3 |
| Phoenix Islands | 0.01 | 0-0.21 | 0 | 0 |
| Johnston Atoll | 0 | 0 | 0 | 0 |
| Wake | 0.61 | 0-1.83 | 0.464 | 0-2.8 |
| Hawaiian Islands | 0.069 | 0-1.4 | 0.23 | 0-16.7 |
| **Overall average prevalence (%)** | **0.14** |  | **0.21** |  |
